# Nurse worry as a trigger for rapid response team activation improving outcomes: a retrospective cohort study in non-critical units

**DOI:** 10.1186/s12912-024-02645-x

**Published:** 2025-01-13

**Authors:** Luana L.S. Gentil, Milena S. Nascimento, Michele Jaures, Leonardo P. de Carvalho, Claudia R. Laselva, Simone Brandi

**Affiliations:** 1 Departamento de Clínica Médica-Cirúrgica, Hospital Israelita Albert Einstein, São Paulo, Brazil; 2 Departamento de Práticas Assistenciais, Hospital Israelita Albert Einstein, Avenue Albert Einstein, 627-701, São Paulo, 05651-901 Brazil

**Keywords:** Clinical observation, Rapid response team, Nurse worry

## Abstract

**Background:**

Patients hospitalized outside of monitored environments may experience sudden clinical worsening requiring transfer to the Intensive Care Unit. Early detection based on the clinical nurse’s identification of the risk of clinical deterioration represents an opportunity to prevent serious adverse events. Nurse worry is defined as the use of clinical reasoning combined with intuition that precedes the patient’s clinical deterioration.

**Objective:**

The objective of this study was to evaluate nurse worry as a trigger for rapid response team activation in patients hospitalized in non-critical units and its association with the need in ICU admission.

**Methods:**

This retrospective cohort study utilized data retrieved from an anonymized institutional database used to monitor the actions of the rapid response team. Data collected from January 2021 to December 2022 were analyzed, encompassing patients over 18 years old admitted to non-critical units and evaluated by the rapid response team. Analyzed variables included demographic characteristics, MEWS score, and causes for activating the rapid response team, such as changes in vital signs and nurse worry. Main outcomes assessed were transfer to the ICU, medical procedures, and drug administration. Patients were divided into three groups for analysis: those triggered for RRT assessment exclusively by changes in vital signs, those triggered exclusively by nurse worry and those triggered by the nurse worry combined with changes in vital signs.

**Results:**

A total of 4634 rapid response team consultations were included, with 1574 triggered by changes in vital signs, 1263 triggered by nurse worry and 1797 triggered by the nurse worry associated with changes in vital signs. The group with nurse concern showed a lower need for transfers to the ICU (40%) compared to the group with changes in vital signs (50%) *p* < 0.001 although there was no difference in relation to the need for medical procedures,17% in both groups.

**Conclusion:**

The NW emerges as a relevant factor in triggering RRT and may be associated with improved outcomes, such as reduced need for ICU transfers. However, the observational design of the study does not allow for establishing causal relationships.

## Introduction

 Patients admitted outside the intensive care unit may experience sudden clinical worsening and require transfer to intensive care units (ICUs) or, in more severe cases, even cardiorespiratory arrest and death. When clinical deterioration is ongoing, the onset of a more severe condition is usually preceded by changes in physiological parameters [1, 2]. Studies show that signs of physiological deterioration can occur from minutes to 8 h before a critical event [1, 3, 4].

Early detection and intervention in situations of clinical instability represent an opportunity to prevent cardiorespiratory arrest in these patients and increase the safety of hospitalization [5].

In this context, the concept of the Rapid Response Team (RRT), a generally multidisciplinary team that can identify hospitalized patients at risk of clinical deterioration early, has been proposed to improve the safety of patients outside the intensive care setting [6, 7].

Although RRT is based on the fundamental principle that early intervention can prevent avoidable morbidity and mortality in a non-critical hospital setting, the evidence on its direct impact on reducing mortality is still debated in the literature [8]. Despite this, several studies suggest that RRT contributes to the early detection of clinical deterioration and rapid intervention, which may improve other important clinical outcomes, such as reducing unplanned transfers to the ICU and length of hospital stay [9–11].

Despite the lack of a universal standard, most triggering criteria include physiological abnormalities, such as respiratory rate, heart rate, systolic blood pressure, oxygen saturation, altered level of consciousness and urine output. The additional criterion may include a team member or family member concerned about the patient’s condition or uncontrolled pain [7, 12].

The literature has indicated that an important cause of triggering RRT is the concern of nurses, which is defined as a criterion for nurses to call for assistance when other criteria have not yet reached a triggering threshold for RRT [13].

Often, the nursing team is the first to identify clinical changes in patients. The clinical perception of nurses regarding the subtle changes in the physiological signs of patients may be an important trigger for triggering RRT, enabling rapid intervention and reducing the chances of serious complications, improving the safety and quality of care provided.

## Objectives

The objective of this study was to evaluate nurse worry as a trigger for rapid response team activation in patients hospitalized in non-critical units and its association with the need in ICU admission.

## Methods

### Study type and location

A retrospective cohort study was performed at a large private quaternary-level hospital. This study followed the STrengthening the Reporting of OBservational studies in Epidemiology (STROBE) for observational studies.

The institutional RRT comprises an intensivist hired by the ICU and assigned to evaluate patients outside the intensive care setting. The RRT service level involves arriving at the location of the code service within 5 min after activation.

### Ethical aspects

The project was approved by the Research Ethics Committee of the Hospital Israelita Albert Einstein, number 70986923.4.0000.0071, and authorized to be exempted from signing the Free and Informed Consent Form.

### Inclusion criteria and exclusion criteria

Patients older than 18 years old admitted to non-critical units whose RRT was activated were included in the study. Patients who passed through emergency care units, intensive care units or pediatrics and whose data were incomplete were excluded.

### Data collection

All study data were retrieved from an anonymized database monitored by the Department of Healthcare Practices. For this study, the data collected during the period from January 2021 to December 2022 were utilized. The department in which the study was carried out admitted around 72 thousand patients during this period.

### Clinical variables

For demographic characterization, variables such as age, gender, MEWs score 6-hours before action, activation time (early or night shift) and time between admission and RRT activation were evaluated. The Modified Early Warning Score (MEWS), which includes an assessment of heart rate, respiratory rate, systolic blood pressure, temperature and level of consciousness, was utilized to assess clinical severity in patients hospitalized outside the intensive care setting [14, 15].

The reasons for RRT activation were also identified and included peripheral oxygen saturation (SpO2) < = 90%, respiratory rate (RR) < 8 or > 28 respiration/minute, systolic blood pressure (SBP) < 90 or > 180 mmHg, heart rate (HR) < 40 or > 130 beats/minute, changes in the level of consciousness and nurse worry for the patient’s condition. Nurse worry is defined as the use of clinical reasoning combined with intuition that precedes the patient’s clinical deterioration. Intuition is defined as a judgment without a rationale, a direct apprehension and response without recourse to calculative rationality [16].

The outcomes assessed in this study included transfer to the ICU and/or any procedures such as venipuncture, urinary catheterization, lab exams or medications prescribed as a result of RRT activation.

### Planning of statistical methods

The data are presented as absolute and relative frequencies for categorical variables and as means, standard errors (SEs) or medians and quartiles, in addition to minimum and maximum values for numerical variables. The distributions of the numerical variables were studied using histograms, boxplots, graphs of comparisons of quartiles and Shapiro‒Wilk normality tests.

For the data analysis, the patients evaluated by the RRT were classified into three groups:

- Abnormal vital signs group (AVS-only): This group included patients who had RRT activation exclusively due to presented alterations in one or more vital signs or level of consciousness.
- Nurse Worry group (NW only): This group included patients who had RRT activation exclusively due to nurse worry reason.
- Nurse Worry and abnormal vital signs (NW & AVS): This group included patients who had RRT activation due to nurse worry and vital signs abnormalities.

Comparisons of variables, both clinical and non-clinical variables were analyzed among the three groups using chi square test for categorical variables and ANOVA for parametric continuous variables or the Kruskal‒Wallis test for nonparametric continuous variables. Multiple logistic regression was used to analyze the ability of the MEWS to predict the admission of patients to the ICU. R software was used for data analysis, and Prism Plus 9 and Wizard 2 were used for graphical analysis.

## Results

Considering the hospital database, 4643 cases RRT activation were included for evaluating the RRT, nine activations were excluded due to incomplete data.

**Fig. 1:**
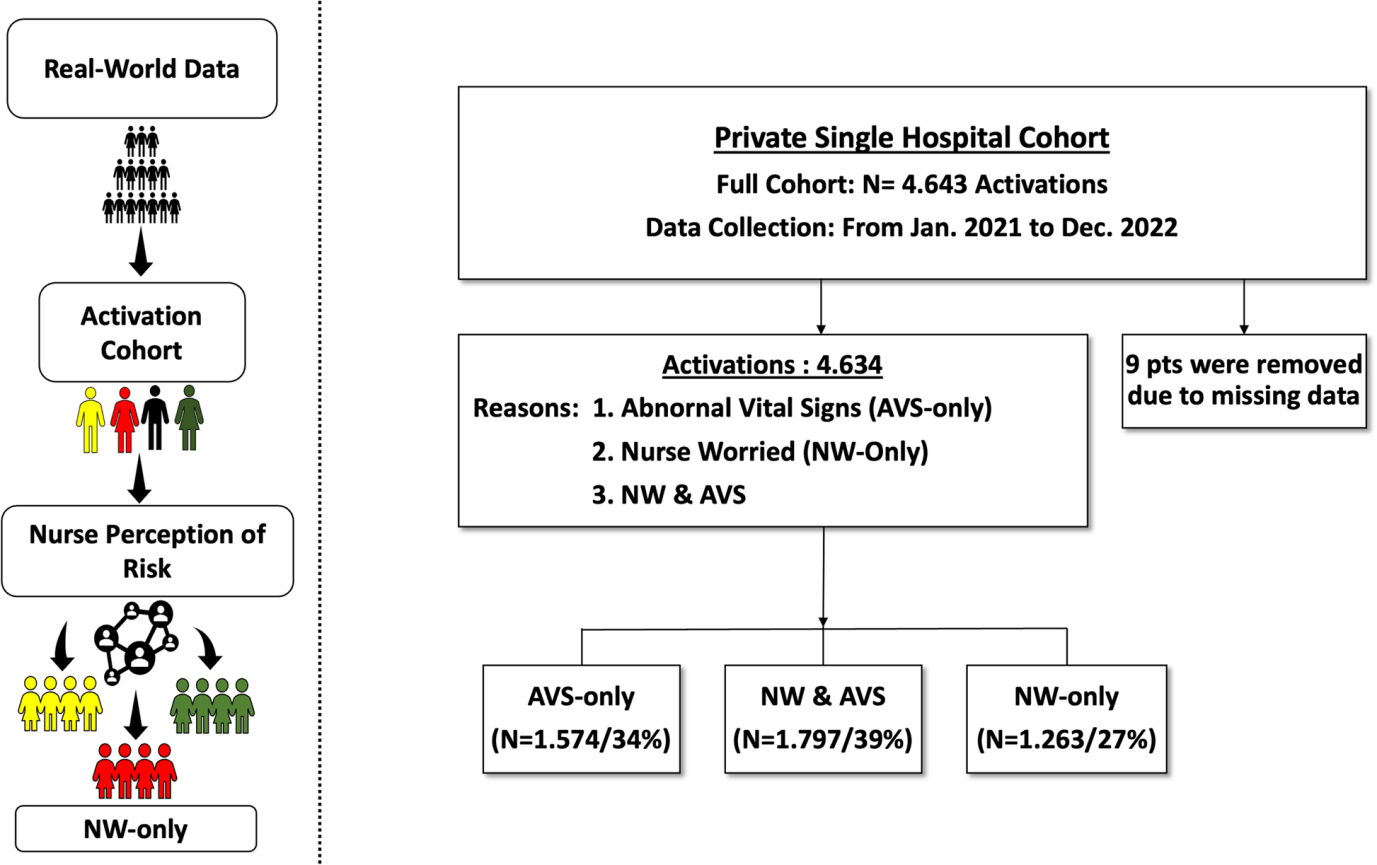
Flowchart representing the distribution of RRT activations in the Sample. NW-only: Total number of activations caused by a nurse perception of risk. AVS-only: RRT activations performed exclusively by abnormal vital signs. NW & AVS: RRT activations carried out due to the nurse worry associated with changes in one or more vital signs. NW-only: RRT activations carried out exclusively by the nurse worry

The sample consisted of 4634 RRT activations, of which 1.574 activations (34%) were triggered by AVS-only group, 1.797 (39%) in NW & AVS group and 1.263 (27%) in NW-only group (Fig. 1). The demographic characteristics of the patients are described in Table 1. The NW-only group had the younger age (60-y ± 30) population (*p* < 0.001) with lower mean time between admission to activation 12 ± 1 days (*p* < 0.001) in comparison to other groups. The mean MEWs score collected 6-h before activation were low and not different among groups: 2.3 ± 1.5 (AVS-only), 2.2 ± 1.5 (NW & AVS) and 2.4 ± 1.5 (NW-only). There was an equipoise time of RRT activation among the three groups being 47%, 52% and 50% at night shift.

**Table 1:** Demographic characterization of the 4634 rapid response time activations

	AVS-only	NW & AVS	NW-only	*p*-value*
**RRT Activations (N)**	**1.574**	**1.797**	**1.263**	
**Gender; n (%)**				
Female	782 (50%)	846 (48%)	669 (53%)	0.04
**Age; Years**				
Mean ± SD	62 ± 30	66 ± 20	60 ± 30	<0.001
**Activation Shift (Night)**	740 (47%)	934 (52%)	631 (50%)	0.02
**Mean between Admission to Activation (Days)**	14 ± 1	17 ± 1	12 ± 1	<0.001
**Mews Score (6 hours before Activation)**				
Mean ± SD	2.3 ± 1.5	2.2 ± 1.5	2.4 ± 1.5	0.03
**Healthcare Resouces Usage**				
Medication (Drugs)	1.180 (75%)	1.311 (73%)	952 (77%)	0.003
Any Procedure	268 (17%)	449 (25%)	214 (17%)	<0.001
Laboratory Exams	677 (43%)	1.060 (59%)	694 (55%)	<0.001

Data are described using absolute and relative frequencies or mean and standard Error

ANOVA Test; *p-value signiifcant (<0.05)

*RRT *Rapid Response Time, *SBP *Systolic Blood Pressure, *SE *Standard Error)

### Clinical deterioration score (MEWs score)

**Fig. 2:**
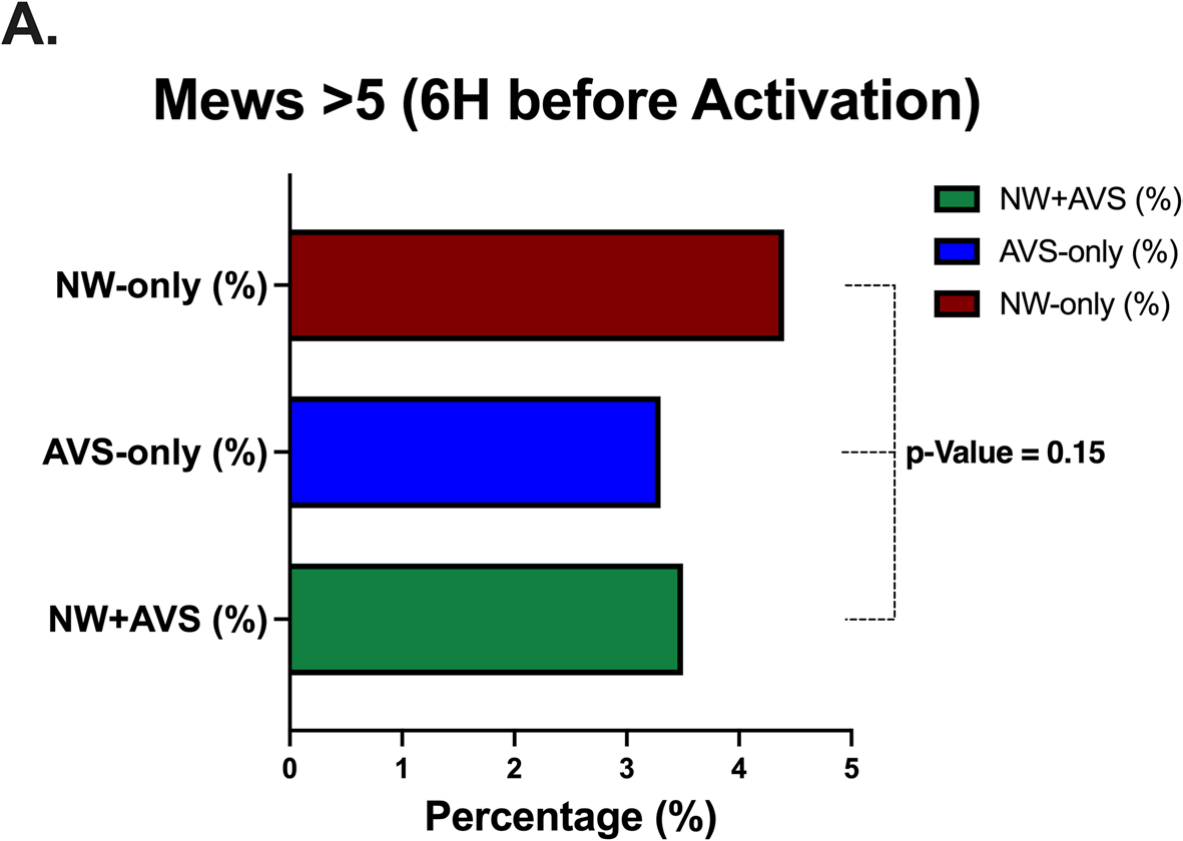
Comparison of the MEWS score > 5 in the last 6-hours prior to RRT activation between the AVS-only, NW & AVS and NW-only groups

Stratified analysis using MEWS > 5 reference in the last 6 h before triggering RRT [17] had the same percentage among groups. AVS-only group had 3,3% of MEWs more than 5, NW-only group had 4,4% and NW + AVS group had 3,5% (*p*-value = 0.15) (Fig. 2).

### Use of healthcare resources

Healthcare resources measured by drug usage, any procedures and lab exams had a particular partner among each group. NW-only had 77,17 and 55%, NW & AVS had 73, 25 and 59% and AVS-only group had 75, 17 and 43% of usage in each group, respectively, all with significant *p*-values (Table 1.).

### ICU transfer

**Fig. 3:**
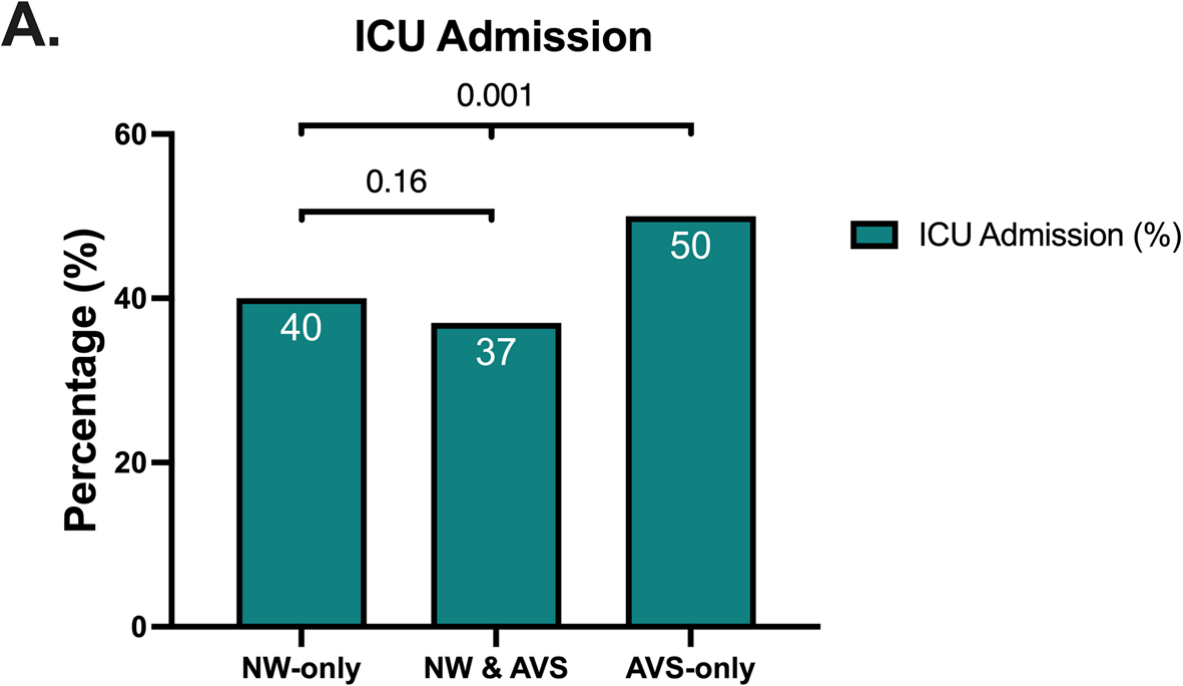
Comparison of healthcare resources usage by admission to ICU among the three studied groups: AVS-only groups, NW & AVS and NW-only

ICU transfer were higher in the AVS-only group (50%) compared to the other two groups: NW-only (40%) and NW + AVS (37%); *p* < 0.001. However no significant difference was observed in ICU admission between NW-only and NW & AVS groups (*p*-value = 0.16) (Fig. 3).

### ICU admission and predictive variables

The MEWs score had an incremental risk of admission with a significant overall *p* < 0.001 (Table 2). In a multivariable analysis using age, gender, NW-only group, MEW score > 5, drug usage, any procedure and laboratory exams all had incremental odds ratio showing higher association with ICU transfer and in contrast if the RRT was triggered by nurse worry only, there was a 57% lower likelihood of transfer to the ICU (*p*-value < 0.001) (Table 3).

**Table 2:**
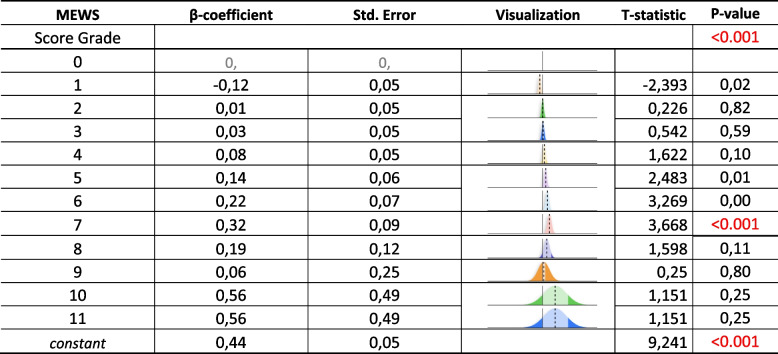
Prediction of ICU admission risk using the MEWS score

**Table 3:**
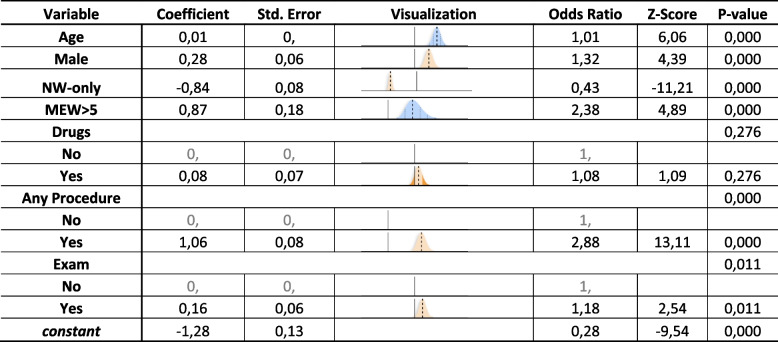
ICU admission predictive variables

## Discussion

Our study’s main finding is that nurses’ role in anticipating the need for patient evaluation by RRT activation is key inpatient safety by reducing patient ICU transfer and healthcare resources usage. Moreover, the NW risk identification was such that this was observed in the group of patients with or without abnormal vital signs.

The detection of clinical deterioration by RRT triggering in hospitalization units of noncritical patients has been widely discussed in the literature, because RRT is some authors conclude as one of the main barriers in hospitalization units to avoid fatal and hospital adverse events [9], however, time of activation is crucial in this process [18]. On the other hand, activation without proper clinical indication unnecessarily requires the use of resources, as it involves the displacement of a multidisciplinary team to evaluate the patient [9, 10]. The existing literature, although supporting the role of RRTs in the early identification of clinical deterioration, still presents conflicting results regarding the impact of these teams on in-hospital mortality. For example, a recent study found no association between the implementation of RRTs and a reduction in in-hospital mortality [8]. These findings reinforce the need for further studies to identify best practices in the implementation and use of RRTs, in addition to exploring the impact of subjective factors, such as the nurse’s clinical judgment, on decision-making.

For this reason, the use of early warning scores to predict early clinical deterioration has emerged as a more effective form of evaluation than the use of isolated vital signs [19–24]. There are numerous scores described in the literature, the most used being the National Early Warning Scale (NEWS) [25] and the Modified Early Warning Scale (MEWS) [15] and recently AI-based systems are being developed that use electronic patient record data and machine learning to predict clinical deterioration [26].

Our study has demonstrated that MEWs score was associated with ICU admission, however, it was not time-sensitive enough since in our population it was in the normal range among all three groups at 6-hours before RRT triggering. This was further supported by a stratified analysis using MEWS > 5, as a cut-off of risk already validated in the literature [27], within 6-hours before code activation which has a very small percentage of patients at risk and has not shown statistically significant difference among groups.

We can conclude that the MEWS score 6 h before RRT activation by itself is not a useful predictor of ICU transfer. The findings suggest that NW may be an important component in the early identification of clinical deterioration, although it is not possible to establish causal relationships due to the observational design of the study.

Accordingly, to Kia et al. study which tested and trained three machine learning models, the comparison among MEWS score and the developed machine learning models has shown that all can alert patients about deterioration 6-hours before the event, providing a basis for timely clinical decisions [28]. The evolution of technology has been providing new tools to support clinical decision-making, and AI been emerging as a promising resource in this context. By integrating large volumes of data, such as vital signs, medical histories, and laboratory parameters, AI helps identify complex patterns [26, 28] This has shown that clinical deterioration detection and RRT activation in non-ICU units is a highly complex process that is influenced by many factors, such as the environment and institution culture, the experience and training of nurses, the relationship between the medical and nursing staff and the belief that early warning plays a vital role in changing outcomes [29–31].

Mohammmed et al. highlighted the possibility of triggering RRT based on abnormal vital signs and emphasized improvements in clinical assessment and decision-making skills [32], our results suggest that triggering RRT by NW, regardless of changes in vital signs, could reduce the need for transfer to the ICU. The importance of NW has been corroborated by two systematic reviews ( [19, 33]). The first review found 25 early warning systems for the recognition of clinical deterioration, seven of which included ‘worry’ as a parameter for triggering the RRT. The inclusion of this parameter allowed the nurse to request help even if the physiological thresholds of a patient were not altered ( [19]). The second review showed that the subjective feeling of nurse concerning was valuable in the process of recognizing patients with clinical deterioration in general wards and that its presence even before the change in vital signs suggested the potential for improving care in an early stage of deterioration [33]. The fact that it is present before changes in vital signs occur suggests the potential for improving care at an early stage of the disease using nurse perception of risk.

Regarding the results of the use of healthcare resources comparison between the 3 groups showed a difference in medication use as well as exams, being higher in the NW-only group. Although this group did not present changes in vital signs, patients required medications and exams, suggesting that the activation of the RRT was not trivialized by the nurse and that patients required intervention for clinical stabilization.

The unplanned transfer of patients to the ICU is associated with greater clinical severity and, consequently, longer hospitalization and higher cost and mortality rate [34, 35] has been considered an important indicator of adverse events [36]. The literature indicates that the main risk factors for unplanned transfer to the ICU are age and the presence of comorbidities [37, 38]. According to our analysis of several variables related to the risk of transfer to the ICU, age, MEWS > 5 and procedure performance due to triggering RRT were found to be independent factors for ICU admission.

Our study has several limitations. First, the study was conducted in a single health care institution, limiting the generalization of the results, which may also influenced by the specific practices of the institution studied, not completely reflecting the diversity of approaches used in other health care settings. Second, the retrospective nature of the study may introduce selection biases and limit the ability to establish cause-and-effect relationships. Third, we used the MEWS score 6 h before RRT activation, but by itself it was not a useful predictor of transfer to the ICU. Fourth, several factors may influence the perception of risk, such as the workload of the nursing team; these factors were not addressed in the present study.

Although clinical deterioration is fundamentally based on changes in vital signs, and using an early warning score to predict clinical deterioration is a useful tool for patient assessment, it alone would not be able to predict risk for escalation of care. One study conducted a comparative analysis of the 5 most used early warning scores, including the MEWS, to assess how they would perform if automated, and the result was that there would be over 1 million false alerts if they were used without critical assessment by a professional [39]. Therefore, the nurse’s clinical judgment and risk perception becomes a valuable critical component in the process of recognizing patients with clinical deterioration even before changes in vital signs, aiding appropriate decision making.

## Conclusion

This retrospective study provides suggestive evidence that nurse worry is an important factor in triggering RRT and may be associated with improved outcomes, such as reduced need for ICU transfers. However, we acknowledge that the observational design of the study does not allow for establishing causal relationships. Therefore, our results highlight the importance of integrating objective tools, such as the MEWS, into clinical judgment, especially in settings where vital signs alone may be insufficient to predict deterioration. Future studies may benefit from the inclusion of multiple institutions and from considering qualitative approaches to better understand the reasons behind nurses’ decisions.

## Data Availability

The datasets used and/or analyzed during the current study are available from the corresponding author on reasonable request.
